# Visual and refractive outcomes following implantation of a new trifocal intraocular lens

**DOI:** 10.1186/s40662-017-0076-8

**Published:** 2017-04-04

**Authors:** Michael Lawless, Chris Hodge, Joe Reich, Lewis Levitz, Uday K. Bhatt, Colm McAlinden, Kate Roberts, Timothy V. Roberts

**Affiliations:** 1 Vision Eye Institute, Level 3 270 Victoria Avenue Chatswood, Sydney, NSW 2067 Australia; 2 Sydney Medical School, University of Sydney, Sydney, NSW Australia; 3 University of Technology, Sydney, NSW Australia; 4 Vision Eye Institute, Melbourne, VIC Australia; 5 University Hospitals Bristol, Bristol, UK

**Keywords:** Trifocal, Trifocal, IOL, Cataract, Refractive lens exchange

## Abstract

**Background:**

Independence from all optical aids, and freedom from unwanted symptoms, following cataract and lens surgery remains the ultimate goal of both patient and surgeon. The development of trifocal IOL technology provides an ever-increasing range of options. The purpose of our study is to understand the predictability, safety and efficacy of a new trifocal intraocular lens (IOL) following cataract or refractive lens exchange (RLE) surgery.

**Methods:**

This was a retrospective consecutive case series of patients undergoing cataract extraction or RLE followed by implantation of the Alcon IQ Panoptix IOL. Pre and postoperative refractive and visual parameters were recorded and evaluated. As the cohort followed a normal distribution, standard parametric tests were used. Paired *t*-test was used to compare the difference between target and postoperative refractive errors. The incidence of intraoperative and postoperative complications was also reported.

**Results:**

The IOL was implanted in 66 eyes of 33 patients. Mean postoperative spherical equivalent (SE) refraction was -0.08 ± 0.25 dioptres (D). This was not significantly different from the target refraction (*p* = 0.841). Sixty-five percent of patients were within ± 0.25 D of the target SE refraction with 100% within ± 0.50 D of intended correction. Mean postoperative uncorrected distance visual acuity (UDVA) was 0.01 ± 0.10 LogMAR. All patients achieved an unaided distance acuity of 20/40 or better postoperatively. Binocularly, 100% saw 0.20 LogMAR or better at near without correction and 88.9% achieved this level for uncorrected intermediate visual acuity. No intraoperative complications were noted. Five patients complained of moderate haloes in the early postoperative period.

**Conclusion:**

The AcrySof IQ Panoptix IOL provides functional uncorrected visual acuity at distance, intermediate and near positions. Our results remain equivalent with existing trifocal IOL outcomes and provide surgeons with a further IOL alternative for the patient motivated to obtain true spectacle independence. Surgeons should consider individual reading and working requirements when counselling patients preoperatively to optimise postoperative patient satisfaction.

**Electronic supplementary material:**

The online version of this article (doi:10.1186/s40662-017-0076-8) contains supplementary material, which is available to authorized users.

## Background

The use of presbyopia correcting intraocular lenses (IOLs) has shown modest growth since their introduction in the 1980s with an approximate 2.4% share of the current global IOL market [1]. High add powers dominated early multifocal models providing excellent near vision. A shift in functional vision requirements however has more recently seen a move towards lower add powers enabling good intermediate acuity (i.e. for use with desktop computers) and augmented distance vision [2, 3]. The recent development of trifocal IOLs provides an expanded range of unaided close vision allowing for further benefits over multifocal IOL predecessors [4–9]. Most available trifocal models utilise a diffractive platform albeit with an emphasis upon slightly different focal points for near and intermediate activities (between 35 to 45 cm and 60 to 80 cm, respectively). These new options provide the ophthalmologist with an opportunity to customise the approach to individual patient requirements.

The Acrysof IQ Panoptix IOL (Alcon Surgical, Inc.) represents the most recent addition to the presbyopia-correcting trifocal IOL market. To our knowledge, we provide the first significant case series of patients undergoing cataract or refractive lens extraction with implantation of the Panoptix IOL describing the visual and safety outcomes in a retrospective, multi-centre cohort.

## Methods

A retrospective assessment of consecutive patients undergoing implantation of the AcrySof IQ Panoptix IOL (Alcon Labs, Ft Worth, TX, USA) at 3 separate centres with 5 doctors (ML, JR, LL, TR, UB) was performed. The indication for surgery included both cataract removal and refractive lens exchange (RLE). All patients were motivated to obtain independence from optical aids following surgery. Patients with significant concurrent ocular disease that would contribute to poor postoperative visual acuity were not considered for the use of the multifocal IOL. Furthermore, the available version of the Panoptix IOL was non-toric, this excluded patients that would have benefited from a toric IOL. Previously, the Panoptix IOL had been approved by the Australian Therapeutic Goods Administration (TGA) regulations agency and use of the IOL was thereby not considered off-label. File notes confirmed that operating surgeons discussed the following with each patient prior to surgery: the risk of vision loss and or surgical complications, the possibility of continued need for optical aids and the presence of optical phenomena such as haloes or glare post-surgery. Surgical consent was obtained in each case before proceeding.

The AcrySof IQ Panoptix IOL specifications and surgery have been described elsewhere [9]. Briefly, the IOL is a 1-piece aspheric hydrophobic IOL. The IOL has a 6 mm optical zone with a central 4.5 mm (15 diffractive zones) and an outer refractive zone to deliver 3 focal points from distance to intermediate and near ranges. Light from the first focal point is diffracted to the distance focus. Optimal close reading distances are provided at 60 cm and 42 cm. The light efficiency of the IOL has been measured at 88% for distance which remains comparable to existing multifocal intraocular lenses [10].

The surgery was performed in each case with topical anaesthesia. A femtosecond laser (LenSx, Alcon Ft Worth, TX, USA) was used to create the capsulorhexis and provide phacofragmentation. Corneal incisions were created manually through a 2.2 mm incision. The company provided “A” constant was used in conjunction with either Holladay II or Barrett Universal II IOL calculation formulas as per surgeon preference (Holladay IOL Consultant, Houston, TX, USA and www.apacrs.org/barrett_universal2 respectively). Refractive targets aimed for the minimum residual myopia. Postoperative medication regimen varied slightly between clinics however, each patient received a combination of antibiotic and anti-inflammatory drops titrated through 4 weeks following surgery.

Patients returned for follow up at 1 day, 1 week, 4 weeks and 2 months. The most recent follow up visit details were recorded. Monocular and binocular uncorrected distance, intermediate (60 cm) and near (40 cm) visual acuities were collected. Visual acuity was converted from Snellen and Revised American Point-Type to LogMAR for analysis. Near and intermediate reading charts were limited to a minimum of N4 (Snellen equivalent approximately 20/25). Intraoperative and postoperative complications were recorded.

Data was collected in Microsoft Excel prior to analysis of basic parameters. The cohort followed a normal distribution therefore, standard parametric tests were used. The paired *t*-test was used to compare the difference between target spherical equivalent (SE) refraction and postoperative SE.

## Results

Sixty-six eyes of 33 patients underwent cataract or clear lens extraction and implantation of an AcrySof IQ Panoptix IOL. Thirty-eight eyes underwent RLE (57.6%). Preoperative demographics are listed in Table 1. The mean length of follow up was 5.7 ± 1.7 weeks (range 4 to 9 weeks). The range of IOL powers used varied between 18.5 D and 27.5 D. The mean preoperative corneal cylinder was 0.50 ± 0.28 D (range 0 to 1.02 D).

**Table 1:** Preoperative characteristics

	Mean (SD)	Min	Max
Sphere (D)	1.66 (1.21)	−1.50	+4.00
Cylinder (D)	−0.50 (0.39)	−1.50	0.00
SE (D)	1.41 (1.21)	−1.75	4.00
Mean keratometry (D)	43.12 (1.25)	40.81	45.39
Corneal Astigmatism (D)	0.46 (0.26)	0.00	1.17
Axial length (mm)	23.33 (0.82)	21.44	24.66
Anterior chamber depth (mm)	3.05 (0.41)	2.16	4.09

### Visual acuity

**Fig. 1:**
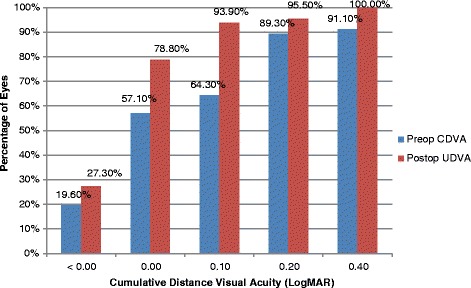
Cumulative Distance Visual Acuity

Mean preoperative corrected distance visual acuity (CDVA) was 0.09 ± 0.20 LogMAR(approximately 20/25). Mean postoperative uncorrected distance visual acuity (UDVA) was 0.01 ± 0.10 LogMAR (approximately 20/20). 78.8% of patients achieved 20/20 UDVA or better. All patients achieved an UDVA of 20/40 or better postoperatively (Fig. 1).

**Fig. 2:**
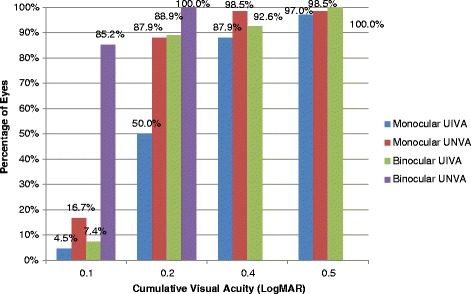
Frequency of Postoperative UIVA and UNVA

The mean uncorrected intermediate visual acuity (UIVA) was 0.30 ± 0.14 LogMAR. Eighty-seven percent (87.9%) of patients achieved 0.40 LogMAR or better at 60 cm monocularly (N8 or J6 equivalent). Fifty percent (50%) of patients achieved 0.2 LogMAR or better (N6 or J2 equivalent) near unaided monocularly with 88.9% additionally achieving this level of vision binocularly at 60 cm at the final visit (Fig. 2). Sixty-three percent (63.6%) of patients achieved 0.14 LogMAR equivalent or better (N4 or J2) for monocular uncorrected near visual acuity (UNVA). Mean LogMAR UNVA was 0.18 ± 0.10. As expected, this increased significantly to 85.2% when both eyes were used together (mean 0.11 ± 0.04 LogMAR) (Fig. 2).

### Refraction

**Fig. 3:**
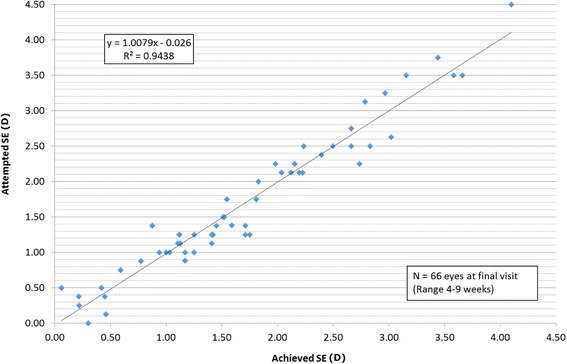
Attempted SE vs. Achieved SE

Mean postoperative SE refraction was -0.08 ± 0.25 D. This was not significantly different from the target refraction (absolute mean difference from target -0.01 ± 0.22 D, *p* = 0.854). Almost two-thirds (65.1%) of patients were within ± 0.25 D of the target refraction with 100% within ± 0.50 D of intended correction. Figure 3 shows the attempted vs. achieved SE (see Additional file 1 for individual vision and refractive outcomes).

At the final postoperative visit, 78.8% of patients achieved UDVA of 0.0 LogMAR or better. Eyes that did not achieve this level had a mean SE of -0.35 ± 0.20 D and mean residual cylinder of -0.45 ± 0.28 D. Those patients achieving 0.0 LogMAR or better had a mean SE of -0.01 ± 0.22 D and mean residual cylinder of -0.09 ± 0.15 D. The difference in SE and cylinder error between groups was statistically significant (*p* = 0.000, 0.000).

### Complications

No intraoperative complications were noted. Five patients complained of moderate glare or haloes through the initial postoperative period following the procedure. This however was not deemed sufficient to impair general activities nor require explant. All patients reported the phenomena diminishing by the subsequent postoperative visit (between 4 weeks to 3 months). There was no tendency to favour either the RLE or cataract group (3 patients/2 patients, respectively). Three patients described having to hold general text closer than prior to surgery, which necessitated minor adjustments to their work environment. Each of these patients confirmed the unaided vision remained more than adequate for their required tasks.

One eye (2.9%) in the RLE group lost 1 line of CDVA at 2 weeks due to drop-related corneal epithelial toxicity however, this resolved by the final visit. Additionally, 20.6% of RLE patients gained 1 line following surgery. Two eyes (7.1%) of one cataract patient did not obtain corrected distance vision (CDVA) of 0.00 LogMAR (20/20) post-operatively. This patient was found to have mild drusen following removal of the cataract that was not apparent prior to surgery. The remaining 92.9% of patients gained between 1 and 6 lines of CDVA.

## Discussion

Presbyopia correcting IOLs represent a significant clinical breakthrough for patients [11]. More recently, the introduction of trifocal IOL models provide surgeons with additional options when considering appropriate IOL selection. Comparative literature highlights the extended reading range provided by the three separate focal points of the trifocal IOLs compared to multifocal IOLs, leading to greater optical independence for all distances [9, 12, 13]. Conversely, the presence of two out-of-focus images increases the likelihood of haloes compared to a single out-of-focus image [14]. Optical bench comparisons between trifocal and multifocal IOLs confirm this albeit with some variation, attributed to IOL design [2, 11, 15–18]. Practical outcomes however, suggest that patient satisfaction remains high despite the increased presence of optical phenomena [19, 20]. Discussing the presence of haloes following trifocal IOL implantation is essential in any preoperative patient discussion.

Our study confirms the predictability and safety of the Panoptix IOL. Our outcomes are consistent with other non-toric trifocal studies. Kohnen previously published the initial findings for the lens reporting the initial outcomes of four patients with the Panoptix IOL. All patients achieved excellent unaided vision at all distances albeit at 1 week follow up. Haloes and glare were present but not considered significant [10]. Separately, Kohnen et al. recently report a mean UDVA of 0.01 ± 0.11 LogMAR with the AtLisa trifocal IOL at 1 and 3 months [21]. Cochener et al. report an UDVA of 0.08 ± 0.11 LogMAR at 3 months following implantation of the FineVision IOL [22]. The follow-up study in 2014 indicated slightly improved results reporting a mean UDVA of 0.01 ± 0.06 LogMAR [5]. Sheppard et al. reported a mean monocular UDVA of 0.19 ± 0.09 LogMAR in a small sample at 2 months with the same IOL [23]. Law et al. found a mean UDVA of 0.05 ± 0.07 LogMAR at 6 months with the AtLisa trifocal IOL [19]. Mojzis et al. report a mean monocular UDVA of -0.03 ± 0.09 LogMAR at 6 months also for the AtLisa IOL [24]. Our results appear broadly equivalent with these findings (0.01 ± 0.10 LogMAR) at a similar postoperative time period. Considering the oft-described process of adaptation, it would be reasonably expected that the unaided vision in our cohort may continue to improve with further follow-up.

The different focal points between models makes direct comparisons of near and intermediate reading values more difficult [9]. The variation in reading tests and their respective limitations similarly impacts comparative references. Considering our results at the measured intermediate and near ranges however, the Panoptix IOL again provides similar findings. Jonkers et al. in a comparative study reported an UIVA and UNVA of 0.32 ± 0.15 and 0.15 ± 0.13 LogMAR, respectively, with the FineVision trifocal IOL [9]. In a small study of 22 eyes, Attia et al. report a mean UIVA and UNVA of 20/20.47 and 20/26.39 respectively, also with the FineVision IOL [6]. Further, Mojzis et al. report equivalent results at the intermediate range with the AtLisa trifocal 0.08 ± 0.10 LogMAR, replicated by Cochener and co-authors in their group with the same IOL model. UNVA in the latter reports were 0.20 ± 0.12 and -0.03 ± 0.04 LogMAR, respectively [5, 24]. Eighty-seven percent (87.9%) of our patients saw 0.20 LogMAR or greater at intermediate whilst 100% achieved this level or better binocularly. Patients who were found to have visual acuity worse than 0.00 LogMAR for distance notably had higher levels of postoperative refractive cylinder emphasizing the importance of limiting residual astigmatism. The use of arcuate incisions represents an option for surgeons, although it is expected a toric version of the trifocal will be available in early 2017 to further assist in minimizing overall postoperative refractive errors.

There were no intraoperative complications within our cohort. Five patients reported moderate haloes initially following surgery. This is consistent with other presbyopia correcting IOLs and with recent bench testing outcomes [25, 26]. Carson et al. suggested that the Panoptix Trifocal IOL showed equivalent or better performance in image quality and resolution compared with alternate trifocal IOLs. Importantly, all patients noted improvement in symptoms over time likely indicating the neuroadaptation process. Further, no patient felt that the haloes impeded their routine activities, suggesting that they were comfortable with the trade-off between symptoms and optical independence. Several patients noted that they had to make adjustments to their standard reading distances. In each case, the patient had slightly more residual myopia than expected, which may have brought forward the respective focal points. This confirms the importance of both accurate biometry calculations and preoperative counselling.

The Alcon Panoptix IOL is based on the same Acrysof 1-piece platform of the single focus IOL. This may have potential advantages over existing models. The stability of this platform has previously been reported [27–29]. Our early refractive outcomes based on the company provided “A” constant suggest that stability and consistency of lens positioning is readily achievable with the new model. In comparison, the AtLisa trifocal IOL is based on a plate-haptic design, which has previously correlated with additional movement within the capsular bag post-surgery [27, 28]. As previously shown across several optical simulations, tilt and decentration may have an impact upon visual performance [29, 30]. This may be exacerbated within presbyopia-correcting IOLs, which demand optimal positioning for maximum effective outcomes. Visual outcomes with the AtLisa trifocal remain excellent however and this putative advantage over plate haptic IOLs remains speculative [31]. The introduction of a toric version of the Panoptix model and further testing may provide additional results to support this hypothesis.

## Conclusions

Our study represents the initial review of a new trifocal IOL. Overall, the IQ Panoptix IOL appears to provide both safety and visual profiles similar to current trifocal models without significant postoperative visual symptoms. Further prospective, case–control studies incorporating additional quality of vision and other subjective traits as well as reading measures may provide more appropriate comparisons with alternate trifocal models [32]. Irrespective of these studies, our results present a further IOL for surgeons to consider in their preoperative assessment with cataract and refractive lens patients who are seeking full optical independence. Particular consideration of patient reading and working requirements is required to optimise the lens model choice.

## Additional file

Additional file 1: Patient pre and post assessment results. (DOCX 27 kb)
