# Supplementary material for: Visual and refractive outcomes following implantation of a new trifocal intraocular lens
Source: Eye Vis (Lond). 2017 Apr 4;4:10. doi: 10.1186/s40662-017-0076-8 (PMC5381044; doi:10.1186/s40662-017-0076-8)
Supplement: Additional file 1: — Patient pre and post assessment results. (DOCX 27 kb) [file 40662_2017_76_MOESM1_ESM.docx]

**Additional file**

**Patient refractive and visual data (n = 66)**

Visual acuity and refractive data at preoperative and final postoperative visits (week visit as marked)

| Eye Number | Pre Sphere (D) | Pre Cylinder (D) | Preop CDVA (LogMAR) | Pre Corneal Cylinder (D) | Pre Mean Keratometry (D) | Postop UDVA (LogMAR) | Post Sphere (D) | Post Cylinder (D) | Postop CDVA (LogMAR) | Post UIVA (LogMAR) | Post UNVA (LogMAR) | Follow up Visit (week) |
| --- | --- | --- | --- | --- | --- | --- | --- | --- | --- | --- | --- | --- |
| 1 | 1.25 | -0.75 | 0.00 | 0.38 | 43.02 | -0.02 | 0 | 0 | 0 | 0.14 | 0.14 | 4 |
| 2 | 1.5 | -0.75 | 0.00 | 0.17 | 43.185 | -0.04 | 0 | 0 | 0 | 0.14 | 0.4 | 4 |
| 3 | 1.25 | -0.25 | -0.06 | 0.28 | 43.46 | -0.06 | 0 | 0 | -0.06 | 0.4 | 0.4 | 9 |
| 4 | 1.25 | 0 | -0.06 | 0.84 | 43.58 | -0.06 | 0 | 0 | -0.06 | 0.4 | 0.4 | 9 |
| 5 | 1.5 | -1 | 0.00 | 0.16 | 41.59 | 0.06 | 0.25 | -0.5 | 0 | 0.4 | 0.14 | 4 |
| 6 | 1 | -0.5 | 0.00 | 0.45 | 41.335 | 0 | 0.25 | 0 | 0 | 0.4 | 0.14 | 4 |
| 7 | 2.25 | -0.5 | 0.00 | 0.28 | 43.41 | 0 | 0 | 0 | -0.06 | 0.14 | 0.14 | 5 |
| 8 | 2.5 | -0.25 | 0.00 | 0.35 | 44.405 | 0 | 0 | -0.25 | 0 | 0.4 | 0.2 | 5 |
| 9 | 1.5 | 0 | 0.00 | 0.99 | 41.955 | -0.02 | 0 | 0 | -0.02 | 0.1 | 0.1 | 4 |
| 10 | 0 | 0 | 0.20 | 0.31 | 41.875 | 0 | 0.5 | 0 | -0.06 | 0.14 | 0.1 | 4 |
| 11 | 1.5 | -0.5 | 0.00 | 0.67 | 45.395 | 0 | 0 | 0 | 0 | 0.5 | 0.2 | 6 |
| 12 | 1 | -0.25 | 0.00 | 0.49 | 45.305 | 0.1 | -0.5 | 0 | 0 | 0.5 | 0.2 | 6 |
| 13 | 1.25 | -0.5 | 0.00 | 0.21 | 42.295 | -0.06 | 0 | 0 | -0.06 | 0.5 | 0.14 | 4 |
| 14 | 1.5 | -0.5 | 0.00 | 0.11 | 42.295 | -0.12 | 0 | 0 | -0.1 | 0.5 | 0.1 | 4 |
| 15 | 1.25 | -0.25 | 0.00 | 0.55 | 43.545 | 0 | 0 | 0 | 0 | 0.4 | 0.1 | 6 |
| 16 | 1 | 0 | 0.00 | 0.66 | 43.41 | 0 | 0 | 0 | 0 | 0.4 | 0.14 | 6 |
| 17 | 1.5 | -1 | 0.00 | 0.43 | 42.775 | 0 | 0 | 0 | 0 | 0.4 | 0.2 | 4 |
| 18 | 1 | -1.25 | 0.10 | 0.3764 | 42.9718 | 0 | 0 | 0 | 0 | 0.4 | 0.2 | 4 |
| 19 | -1.5 | -0.5 | 0.54 | 0.77 | 42.995 | 0.3 | -0.25 | -0.5 | 0 | 0.6 | 0.4 | 4 |
| 20 | 0.5 | 0 | 0.00 | 0.67 | 43.495 | 0 | -0.25 | 0 | 0 | 0.2 | 0.14 | 4 |
| 21 | 2 | -0.25 | 0.00 | 0.21 | 43.35 | -0.04 | 0.25 | 0 | -0.1 | 0.4 | 0.2 | 4 |
| 22 | 2.25 | -0.5 | 0.00 | 0.4 | 43.185 | 0.04 | 0 | -0.5 | -0.1 | 0.2 | 0.14 | 4 |
| 23 | 1.25 | 0 | -0.06 | 0.39 | 43.405 | 0 | 0 | 0 | 0 | 0.4 | 0.2 | 6 |
| 24 | 2 | -0.5 | 0.00 | 0.73 | 43.465 | 0 | 0.5 | 0 | 0 | 0.4 | 0.2 | 6 |
| 25 | 3 | -0.5 | 0.00 | 0.36 | 41.59 | 0 | 0 | 0 | 0 | 0.4 | 0.2 | 5 |
| 26 | 2 | -0.5 | 0.00 | 0.67 | 41.645 | 0 | 0 | 0 | 0 | 0.4 | 0.2 | 5 |
| 27 | 2 | -0.5 | 0.00 | 0.39 | 43.575 | 0 | 0.25 | 0 | 0 | 0.2 | 0.14 | 8 |
| 28 | 0 | -0.5 | 0.50 | 0.58 | 44.35 | 0.1 | -0.5 | 0 | 0 | 0.5 | 0.4 | 8 |
| 29 | 2.75 | -0.5 | -0.16 | 0.06 | 45.21 | -0.16 | 0 | 0 | -0.16 | 0.2 | 0.1 | 8 |
| 30 | 2 | -0.5 | -0.16 | 0.06 | 45.33 | -0.16 | -0.5 | 0 | -0.16 | 0.4 | 0.1 | 8 |
| 31 | 0 | 0 | 0.50 | 0.31 | 41.715 | -0.02 | -0.25 | 0 | -0.02 | 0.4 | 0.1 | 6 |
| 32 | -0.75 | 0 | 1.00 | 0.84 | 41.98 | 0 | -0.25 | 0 | 0 | 0.2 | 0.1 | 6 |
| 33 | 3.5 | -0.5 | 0.20 | 0.61 | 41.415 | 0.1 | 0 | -0.5 | 0 | 0.4 | 0.1 | 4 |
| 34 | 4 | 0 | 0.20 | 0.62 | 41.62 | 0.1 | -0.25 | -0.5 | 0 | 0.6 | 0.1 | 4 |
| 35 | 2.5 | -0.75 | 0.10 | 0.17 | 43.685 | 0.1 | 0.25 | -0.75 | 0 | 0.2 | 0.1 | 5 |
| 36 | 2.25 | -0.5 | 0.10 | 0.56 | 43.27 | 0.1 | 0.25 | -0.75 | 0 | 0.2 | 0.14 | 5 |
| 37 | 2 | -1.25 | 0.00 | 0 | 42.88 | -0.02 | 0.25 | 0 | -0.02 | 0.2 | 0.14 | 6 |
| 38 | 1.75 | -1.25 | 0.00 | 0.38 | 43.02 | -0.04 | 0.25 | 0 | -0.04 | 0.2 | 0.14 | 6 |
| 39 | -1 | -1 | 0.20 | 0.29 | 44.265 | 0.4 | -0.5 | -0.25 | 0 | 0.2 | 0.14 | 8 |
| 40 | -0.25 | -0.75 | 0.20 | 0.35 | 44.465 | 0.3 | -0.25 | -0.5 | 0 | 0.2 | 0.14 | 8 |
| 41 | 1.75 | -0.75 | 0.20 | 0.33 | 42.775 | 0.2 | 0 | -0.75 | 0.08 | 0.5 | 0.2 | 6 |
| 42 | 1.5 | -0.5 | 0.20 | 0.49 | 42.695 | 0.1 | 0.25 | -0.75 | 0.08 | 0.4 | 0.2 | 6 |
| 43 | 3 | -0.25 | 0.00 | 0.82 | 42.97 | 0 | -0.25 | -0.25 | -0.02 | 0.4 | 0.14 | 6 |
| 44 | 3.5 | -0.5 | 0.00 | 0.72 | 43.24 | 0.1 | -0.5 | 0 | -0.02 | 0.2 | 0.14 | 6 |
| 45 | 3.75 | -0.5 | 0.00 | 0.67 | 45.395 | 0 | 0 | 0 | -0.04 | 0.4 | 0.14 | 9 |
| 46 | 3.5 | -0.75 | 0.00 | 0.25 | 45.365 | 0 | -0.25 | -0.25 | 0 | 0.4 | 0.14 | 9 |
| 47 | 2.5 | -0.25 | 0.00 | 0.47 | 42.055 | 0 | 0 | 0 | 0 | 0.14 | 0.14 | 4 |
| 48 | 2.25 | -0.25 | 0.00 | 0.57 | 41.845 | 0 | 0 | 0 | 0 | 0.14 | 0.14 | 4 |
| 49 | 2.5 | -0.75 | 0.20 | 0.53 | 44.735 | 0 | 0.25 | -0.5 | -0.02 | 0.4 | 0.14 | 4 |
| 50 | 3 | -0.75 | 0.20 | 0.18 | 44.91 | 0 | 0.5 | -0.25 | -0.02 | 0.4 | 0.14 | 4 |
| 51 | 2.75 | -0.5 | 0.20 | 1.17 | 41.445 | 0 | 0 | 0 | 0 | 0.2 | 0.14 | 6 |
| 52 | 3.5 | -1.5 | 0.20 | 0.92 | 41.57 | 0 | 0.25 | -0.25 | 0 | 0.2 | 0.14 | 6 |
| 53 | -0.25 | -1.25 | 0.50 | 0.58 | 44.41 | 0 | 0 | -0.25 | 0 | 0.14 | 0.14 | 8 |
| 54 | 0.5 | -0.5 | 0.20 | 0.51 | 43.745 | 0 | -0.25 | 0 | 0 | 0.14 | 0.14 | 8 |
| 55 | 1.5 | 0 | 0.20 | 0.2 | 40.81 | 0 | 0.25 | -0.25 | -0.1 | 0.2 | 0.14 | 8 |
| 56 | 1.25 | 0 | 0.20 | 0.15 | 41.035 | 0 | 0.25 | -0.25 | -0.1 | 0.2 | 0.14 | 8 |
| 57 | -1.5 | -0.5 | 0 | 0.92 | 42.7 | 0 | -0.25 | -0.25 | -0.1 | 0.14 | 0.2 | 8 |
| 58 | -1.5 | -0.25 | 0.1 | 0.96 | 42.35 | -0.04 | 0 | -0.25 | 0 | 0.14 | 0.2 | 8 |
| 59 | 0.25 | 0 | 0.3 | 0.55 | 41.085 | 0 | 0.25 | 0 | -0.1 | 0.14 | 0.14 | 4 |
| 60 | 0.75 | -0.5 | 0.2 | 0.4 | 41.16 | 0 | 0.25 | -0.25 | 0 | 0.4 | 0.14 | 4 |
| 61 | 2.75 | -0.75 | 0.2 | 0.93 | 44.355 | -0.02 | 0 | -0.25 | 0 | 0.4 | 0.6 | 5 |
| 62 | 3 | -0.75 | 0.02 | 0.93 | 44.295 | 0 | -0.25 | 0 | 0 | 0.14 | 0.2 | 5 |
| 63 | 1.25 | -0.5 | -0.04 | 0.6 | 47.44 | 0 | -0.25 | 0 | -0.1 | 0.14 | 0.2 | 6 |
| 64 | 1.25 | 0 | -0.04 | 1.14 | 47.51 | -0.02 | 0.5 | -0.5 | -0.04 | 0.2 | 0.14 | 6 |
| 65 | 1.5 | -1 | 0 | 0.29 | 43.805 | -0.16 | -0.25 | 0 | -0.16 | 0.1 | 0.4 | 4 |
| 66 | 2.25 | -0.75 | 0.04 | 0.22 | 43.21 | -0.16 | 0 | -0.5 | -0.16 | 0.1 | 0.4 | 4 |
